# Enhancing surgical object detection in laparoscopic cholecystectomy with explicit positional relationship modeling

**DOI:** 10.1016/j.csbj.2025.07.056

**Published:** 2025-08-05

**Authors:** Yinan Xu, Yutong Ban, Yue Zhao, Dolores Krauss, Christiane Bruns, Jennifer Eckhoff, Hans Fuchs

**Affiliations:** a Department of General, Visceral and Cancer Surgery, University Hospital of Cologne, Cologne, Germany; b UM-SJTU JI, Shanghai Jiao Tong University, Shanghai, China

**Keywords:** Surgical object detection, Medical imaging, Laparoscopic cholecystectomy, Detection, transformer, Relation modeling

## Abstract

Laparoscopic Cholecystectomy (LC) is one of the most performed complex surgeries. Integrating Artificial Intelligence (AI) into LC shows great potential for assisting in anatomical structure detection. To be dependable, AI must be accurate, robust, and effective. In this study, a relation-based model was proposed to enhance surgical object detection in LC images. The model employs a positional relation encoder and refines progressive attention mechanism to analyze object relationships. Two widely used LC datasets were selected to validate the proposed model. We strictly followed the official split and evaluator protocols for fair comparison with benchmark models. The Macroscopic Correlation (MC) results revealed distinct differences in position relation strength between the two datasets, enabling comprehensive evaluation of the proposed models under different circumstances. The experimental results demonstrated the accuracy and effectiveness of the proposed models in both datasets. The proposed model outperformed the best-performing benchmark model by an improvement of 33.95 % in overall mean Average Precision (AP) on the Endoscapes dataset. For classes Cystic Plate and HC Triangle, the detection AP was improved by 90.32 % and 92.46 %, respectively. For the m2cai16-tool-locations dataset, the proposed models also demonstrated effective performance, improving the overall mAP by up to 17.97 % compared to benchmark models.

The experimental results proved the accuracy and effectiveness of the proposed model. Due to the analysis of position relation, the detection of key objects is significantly improved. The postprocessing steps effectively reduce redundant bounding boxes by over 90 %. Future work could focus on expanding to more clinical and practical applications.

## Graphical abstract

**Figure:**
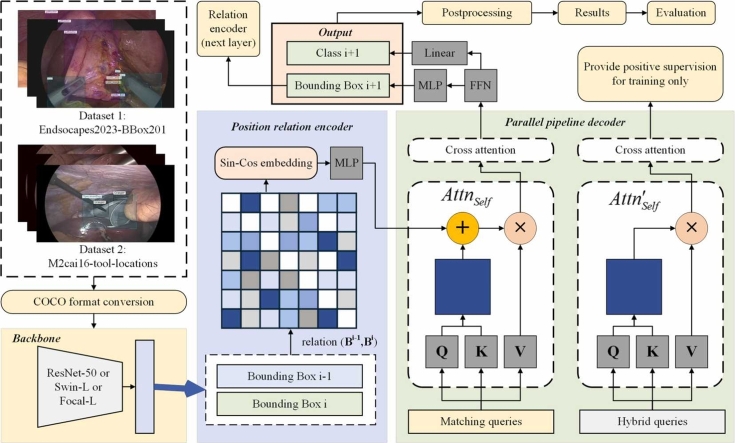

## 1 Introduction

The application of Artificial Intelligence (AI) in medical imaging has demonstrated significant potential [1], [2], [3], [4]. AI can enhance diagnostic accuracy, optimize clinical workflows and assist in decision-making across a wide range of clinical domains [5], including radiology [6], [7], [8], [9], oncology [10], [11], surgery [12], [13], [14], etc. Despite its promise, the widespread clinical adoption of AI remains challenging due to concerns about trust, reliability and the practical integration of AI systems into real-world clinical settings [15], [16]. Establishing trust in AI for medical imaging is a multifaceted challenge, demanding not only high accuracy but also explainability, robustness and validation in varied clinical environments [17]. Addressing these challenges may enable AI to improve surgical guidance, strengthen patient safety and then support its reliable integration into routine clinical practice.

Laparoscopic Cholecystectomy (LC) is one of the most performed minimally invasive surgical procedures [18], primarily used to treat gallbladder diseases such as cholelithiasis and acute cholecystitis [19]. Due to its complexity, LC demands surgical precision and timely intraoperative decision-making [20]. Integrating AI into LC offers considerable promise by improving key procedural components such as intraoperative guidance and precise anatomical structure recognition. Initial research on the application of AI in LC mainly concentrated on modeling surgical phase recognition [21], [22], [23], [24] due to the standardized workflow and high frequency of execution. However, surgical phase recognition provides only a general overview of LC procedures, without addressing the detailed detection and localization of critical anatomical objects required for effective intraoperative support.

In recent years, a growing number of studies have explored the application of AI to more clinical research in LC. For example, the Critical View of Safety (CVS) approach [25] plays a crucial role in ensuring the safety of LC to prevent the Bile Duct Injury (BDI) [26]. Researchers have concentrated on the automatic assessment of CVS in LC to support surgeons with timely and informed decision-making during the procedure [27], [28], [29]. Beyond anatomical structures such as the Cystic Duct, Cystic Artery and Gallbladder in LC images, various surgical tools also provide essential context for understanding the procedure. Initially, researchers concentrated on identifying the presence of various surgical instruments in LC, such as Graspers, Hooks and Bipolar [30], [31], [32], [33]. Subsequently, research shifted towards the detection or segmentation of surgical instruments in images to provide more precise and accurate identification during LC procedures [34], [35]. In addition, combining the surgical tools, motions and anatomical targets into a triplet (instrument, verb, target) has enabled more comprehensive understanding and prediction of surgical workflow in LC [36], [37], [38]. Moreover, AI can contribute to the education and standardization of LC procedures, such as assessing operative skills [34] and supporting AI-assisted coaching programs [39]. However, establishing trust is essential for the effective integration of AI into clinical LC [40]. As a result, achieving high levels of accuracy, reliability, robustness, and effectiveness in AI algorithms is critical to ensure that clinicians can confidently rely on AI-based tools during LC.

This study presents a relation-based model to achieve more accurate and efficient detection of surgical objects in LC images, building upon existing structures [41], [42]. The proposed model incorporates a position relation encoder and a refined progressive attention mechanism to effectively capture and exploit the positional relationships among objects in LC images. A parallel relation streaming pipeline was utilized to help the insufficient positive supervision and duplication removal in object detection. Additionally, a postprocessing step was applied to reduce redundant bounding boxes, thereby ensuring the predictions align closely with clinical realities and surgical practice. To validate the accuracy and effectiveness of the proposed model, experiments were conducted on two widely used public LC datasets [34], [43], [44]. The experimental results demonstrated the accuracy and effectiveness of the proposed models across datasets with varying characteristics. By integrating position relation analysis and a robust postprocessing strategy, the models contribute to building trust in AI with improved accuracy while closely reflecting the clinical realities. Furthermore, to better support clinicians and endoscopists in utilizing our model, we developed a web-based application for easy access and deployment (https://github.com/xyn-abc/LCSOD-tool).

## 2 Methods

### 2.1 Datasets

The public dataset *Endoscapes* [44], provided by ICube at the University of Strasbourg, France is utilized in this study to evaluate the performance of the modified method. In total, 201 LC videos with 58813 frames were collected for CVS research from the University Hospital of Strasbourg. Among 58813 frames, a subset *Endoscapes-BBox201* with 1933 frames (1 frame every 30 s) was annotated by 3 experts with bounding boxes for 6 different classes: Cystic Plate, Hepatocystic Triangle Dissection (HC Triangle), Cystic Artery, Cystic Duct, Gallbladder and Tool. The *Endoscapes-BBox201* is relevant to the surgical sections and anatomical structures of CVS, making this dataset suitable for the object detection assessment in this study. The annotations of *Endoscapes-BBox201* were formatted in the Common Objects in Context (COCO) style. We followed the official subset split from the paper [44]: 1212 frames for training, 409 frames for validation, and 312 frames for testing.

To ensure a comprehensive evaluation, another public dataset *m2cai16-tool-locations* [34] from the Human-Centered Artificial Intelligence, Stanford University is utilized in the study. Based on the M2CAI 2016 Tool Presence Detection Challenge (*m2cai16-tool*) [31], which is a dataset of 15 LC videos annotated with binary labels indicating the presence of the surgical tools, *m2cai16-tool-locations* extends the *m2cai16-tool* with the bounding box annotations of 7 tools in the LC videos, including Grasper, Bipolar, Hook, Scissors, Clipper, Irrigator and Specimen bag. In *m2cai16-tool-locations,* a total of 2811 frames are annotated with the bounding boxes and class labels of the tools under supervision and spot-checking by an expert surgeon. In this study, we followed the official subset split from the paper [34]: 50 % (1405 frames), 30 % (843 frames) and 20 % (563 frames) for the training set, validation set and testing set, respectively.

### 2.2 Evaluation metrics

In this study, the Macroscopic Correlation (MC) [41] is employed to statistically assess the position relationship among bounding boxes in LC images. MC was proposed based on the Pearson Correlation Coefficient (PCC) [45]. The PCC between each pair of bounding boxes is computed and utilized as the corresponding edge weight. The MC can be calculated by the graph intensity with the Eq. (1):

(1) $$MC=\frac{\sum_{a}\sum_{b:a\neq b}\left|PCC\left(B_{a},B_{b}\right)\right|}{N\left(N-1\right)}$$

where N is the number of annotated bounding boxes in the image. B=[x,y,w,h] is the annotation of each bounding box in the image. MC∈[0,1] represents the degree of the position relationship of the features in the image. The closer the MC value is to 1, the stronger the position correlation between the bounding boxes. According to the common interpretations of the strength of correlation coefficients in statistics [45], 0.0–0.3 MC is considered low or no position relationship correlation, 0.3–0.5 MC indicates a moderate position relationship correlation and 0.5–1.0 MC represents a strong position relationship correlation.

Standard evaluation metrics for object detection were employed to assess the performance of the Relation-LCSOD models. mAP was used as the primary metric to evaluate the overall performance across all categories in the two datasets. The mAP was calculated as follows in Eq. (2):

(2) $$\left\{ \begin{array}{l} mAP=\frac{\sum_{i=1}^{N}AP}{N}\\ AP=\int_{0}^{1}P\left(R\right)dR \end{array}\right.$$

where N is the number of categories in the dataset. AP is the Average Precision (AP) for each category. P(R) is the precision-recall curve, where precision=TP/(TP+FP) and recall=TP/(TP+FN). In object detection, the number of True Positive (TP), True Negative (TN), False Positive (FP) and False Negative (FN) cases are determined by the Intersection over Union (IoU). IoU measures the overlap between the predicted bounding box and the ground truth, defined as the ratio of the intersection area to the union area of the two bounding boxes. The ground truth refers to the bounding boxes manually annotated by endoscopists, which serve as the reference standard for evaluation. A higher IoU threshold imposes stricter criteria for determining a TP case, requiring the predicted bounding box to exhibit greater overlap with the ground truth. Following standard practices in object detection literature, evaluation metrics were calculated across various IoU thresholds in this study, ranging from 0.50 to 0.95 with an interval of 0.05.

### 2.3 Model architecture: Relation-LCSOD

**Fig. 1:**
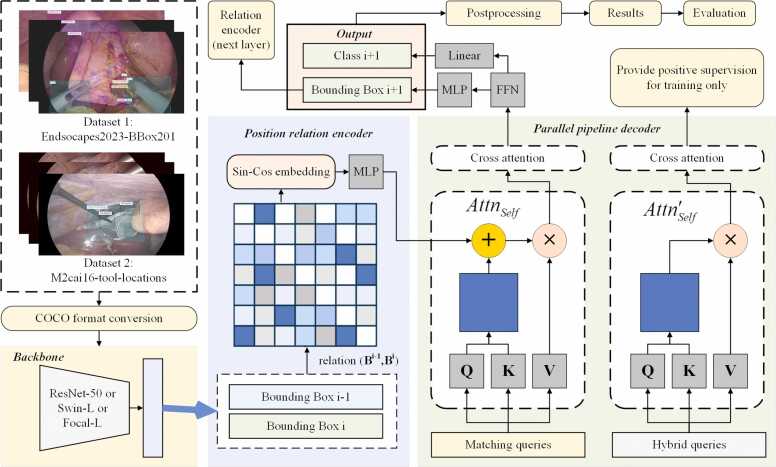
The framework of the Relation-LCSOD model in the study. ResNet-50, Swin-L and Focal-L are selected as the backbones for feature extraction. The position relation relation(B,B) is embedded in the position relation encoder by. A parallel pipeline is utilized for the decoder structure, where refined progressive attention $Attn_{Self}$ is proposed for matching queries.

Hou et al. proposed a Relation-DETR structure, enhancing the traditional DETR with explicit position relation prior [41]. We further refine the model with data processing and results postprocessing to enhance its suitability for detecting surgical objects in LC, named Relation-based Laparoscopic Cholecystectomy Surgical Objects Detector (Relation-LCSOD). In Relation-LCSOD, the traditional encoder and decoder of DETR are enhanced with explicit positional relationship information. The structure of Relation-LCSOD is illustrated in Fig. 1 and described in the following sections. The DETR encoder and decoder structures are both enhanced by the relation modification, including position relation encoder, refined progressive self-attention and parallel relation pipeline. The *m2cai16-tool-locations* dataset is transformed into the standard COCO format for the training and testing of the proposed method. Additionally, adjustments are made to further align the number of predictions with the ground truth for each category in the two LC datasets. For categories Cystic Plate, HC Triangle, Cystic Artery, Cystic Duct and Gallbladder in the *Endoscapes-BBox201* dataset*,* only the predictions with the highest scores are retained. For the Tool category in the *Endoscapes-BBox201* dataset, the top 2 predictions with the highest scores are kept. For each category in the *m2cai16-tool-locations* dataset except Grasper, only the predictions with the highest scores are retained. The top 2 predictions with the highest scores for Grasper are kept in the *m2cai16-tool-locations* dataset.

#### 2.3.1 Position relation encoder

In Relation-LCSOD, a position relation encoder is employed to explicitly model the position relationship information between objects. Building upon the DETR encoder structure [46], a high-dimensional positional relation embedding is integrated to the encoder. This module aims to generate embeddings that capture the position relationship of objects, which can enhance the model convergence speed and reduce reliance on large data volumes [41]. Relative geometry features between different bounding boxes are defined in Eq. (3) and then embedded to the encoder by sine-cosine encoding in Eq. (4):

(3) $$e\left(B_{a},B_{b}\right)=\left[\log\left(\frac{\left|x_{a}-x_{b}\right|}{w_{a}}+1\right),\;\log\left(\frac{\left|y_{a}-y_{b}\right|}{h_{a}}+1\right),\;\log\left(\frac{w_{a}}{w_{b}}\right),\;\log\left(\frac{h_{a}}{h_{b}}\right)\right]$$

(4) $$\left\{ \begin{array}{l} \mathrm{Embed}\left(E,2k\right)=\;\sin\left(\frac{sE}{T^{2k/d}}\right)\\ \mathrm{Embed}\left(E,2k+1\right)=\;\cos\left(\frac{sE}{T^{2k/d}}\right) \end{array}\right.$$

where B=[x,y,w,h] is the predicted bounding boxes from decoders. $E\in {\mathbb{R}}^{N\times N\times 4},E\left(a,b\right)=e\left(B_{a},B_{b}\right)$ is the relation matrix. T,d are parameters for encoding. The relation embedding then goes through a linear transformation to generate scalar weights for attention heads in Eq. (5):

(5) relation(B,B)=max(ε,W⋅Embed(B,B)+A)

where ε is a constant to keep the elements in the relation positive, thereby preventing gradient vanishing issues in subsequent calculations. The dimension of relation is N×N×M. Other components in the position relation encoder remain the same as the encoder structure in the traditional DETR, including the backbone for feature extraction, positional encoding, self-attention layers and Feed Forward Network (FFN).

#### 2.3.2 Refined progressive attention

In Relation-LCSOD decoders, the self-attention of each layer not only relies on the query of the current layer, but also refers to the relation information of the previous layer. The formula for self-attention is described in Eq. (6):

(6) $$Attn_{Self}\left(Q^{l}\right)=\mathrm{Softmax}\left(\mathrm{relation}\left(B^{l-1},B^{l}\right)+\frac{Que\left(Q^{l}\right)Key{\left(Q^{l}\right)}^{T}}{\sqrt{d_{model}}}\right)\cdot Val\left(Q^{l}\right)$$

where $Q^{l}$ is the queries of the decoder layer l. Like the DETR, the next layer output can be generated by the following formulas in Eq. (7):

(7) $$\left\{ \begin{array}{l} Q^{l+1}=FFN\left(Q^{l}+Attn_{cross}\left(Attn_{self}\left(Q^{l}\right),Key\left(M\right),Val\left(M\right)\right)\right)\\ B^{l+1}=MLP\left(Q^{l+1}\right)\\ c^{l+1}=Linear\left(Q^{l+1}\right) \end{array}\right.$$

where M is the memory, FFN,MLP,Linear are Fead Forward Network, Multi-Layer Perceptron network and Linear function layer, which are the same as the components in the DETR decoder structure [46]. $B^{l+1},c^{l+1}$are the bounding boxes prediction and class prediction of the next layer.

#### 2.3.3 Parallel relation pipeline

The DETR decoder utilizes a one-to-one matching strategy for object queries, whereby each query is assigned to only one positive sample. This leads to the limitation of the number of positive samples, resulting in the model being unable to obtain sufficient positive supervision during training [41]. Additionally, multiple queries may predict the same target, resulting in duplicate detection problems. Existing duplication removal methods, like Non-Maximum Suppression (NMS) [47], fast-NMS [48] and Soft-NMS [49], cannot solve the duplicate prediction in the end-to-end DETR structure [41]. In Relation-LCSOD, a parallel relation streaming pipeline is employed to overcome insufficient positive supervision and duplication removal. The object queries pipeline in DETR is expanded to two parallel sets of queries: matching queries and hybrid queries. The matching queries $Q_{match}$ go through the self-attention function in Eq. (5), while the hybrid queries $Q_{hybrid}$are processed with the normal self-attention function in Eq. (8):

(8) $$Att{n\prime }_{Self}\left(Q^{l}\right)=\mathrm{Softmax}\left(\frac{Que\left(Q^{l}\right)Key{\left(Q^{l}\right)}^{T}}{\sqrt{d_{mod\;el}}}\right)\cdot Val\left(Q^{l}\right)$$

By skipping the position relation component in the hybrid query stream, the model can generate more diverse potential predictions, which helps to alleviate the issue of insufficient positive supervision. For matching queries using the one-to-one matching strategy, the normal loss function in Eq. (9) is employed to enforce the non-duplicate predictions:

(9) $$\left\{ \begin{array}{l} Loss\left(p_{match},g\right)=\sum_{l=1}^{L}\mathrm{Hungarian}\left(p_{match}^{l},g\right)\\ Loss\left(p_{hybrid},g\right)=\sum_{l=1}^{L}\mathrm{Hungarian}\left(p_{hybrid}^{l},\tilde{g}\right) \end{array}\right.$$

where $p_{match}^{l}=\left(B_{match}^{l},c_{match}^{l}\right)$ is the predictions of the matching queries pipeline. g is the ground truth. For the predictions of the hybrid queries pipeline $p_{hybrid}^{l}=\left(B_{hybrid}^{l},c_{hybrid}^{l}\right)$, the one-to-multiple matching strategy is used. Therefore, the ground truth is a set of repeats for K times $\tilde{g}=\left\{g^{1},g^{2},\ldots ,g^{K}\right\}$. Besides, the predictions of the hybrid queries pipeline are utilized only in training, but not for inference. In this way, positive supervision can be enhanced but duplicate predictions can be avoided to some extent.

## 3 Experimental results and discussion

### 3.1 Experimental design

In this study, all experiments were conducted on a Linux-based platform with a Nvidia RTX 3090 Super GPU (24 GB graphics memory). In Relation-LCSOD, the pre-trained Relation-DETR was finetuned with the Dataset A: *Endoscapes-BBox201* and Dataset B: *m2cai16-tool-locations* and its results were further processed for the LC surgical object detection. ResNet-50 [50], Swin transformer large (Swin-L) [51] and Focal modulation network large (Focal-L) [51] were selected as the backbone for feature extraction. Those backbones were pre-trained on ImageNet [52] and then finetuned with COCO2017 [53], CSD [54] and MSSD [55] datasets during the training of the Relation-DETR encoder and decoder. The Relation-DETR was pre-trained with the following parameters [41]: scheduled learning rates 10^−5^ with step factor 0.1, parameters T,d,s are 10000, 16 and 100, and VariFocal loss [56]. We finetuned the pre-trained Relation-DETR using the two LC datasets for LC surgical object detection to develop a Relation-LCSOD model. Three variations of the model were proposed for surgical object detection for LC images, incorporating three different backbones with the same model parameters: Relation-LCSOD (ResNet-50), Relation-LCSOD (Swin-L) and Relation-LCSOD (Focal-L). 1/2 parameters of the pre-trained model were frozen and then finetuned for 16 epochs with two dataset images using the AdamW optimizer with a scheduled learning rate of 10^−4^ (milestone 12 and gamma 0.1) and weight decay of 10^−4^. The LC dataset images were resized to 256 × 256. The embedding dimension, number of object queries and number of feature levels are set as 256, 900 and 5, respectively. For the parameters of hybrid queries, 1500 bounding boxes generated from hybrid queries are served as candidates and 6 are assigned to each category. The layer number of the encoder and decoder are both set as 6. The number of multi-head attention is set as 8 and the hidden layers dimensions of FFN are set as 2048 in the model. The number of output heads was refined to 7 (6 classes and 1 background) and 8 (7 classes and 1 background) for output compatibility of the *Endoscapes-BBox201* and *m2cai16-tool-locations* dataset respectively. The input images were augmented with random sizing, cropping and flipping using *Albumentations* [57] to increase the sample numbers. Furthermore, the number of output bounding boxes was adjusted to align with the true count of bounding boxes for each category in LC images. We followed the official subset splits of both datasets and utilized their evaluation codes to compare the proposed models versus the benchmark models.

### 3.2 Benchmark models

In order to demonstrate the enhancement of the Relation-LCSOD model, we adopted the official benchmark models outlined in the *Endoscapes-BBox201* technical report [44] and *m2cai16-tool-locations* paper [34] as benchmark models for this study. In the technical report of Murali et al. [44], Faster Reginal-based Convolutional Neural Networks (Faster-RCNN) [58], Cascade-RCNN [59] and Deformable-DETR [60] were officially selected as the benchmark models for the evaluation of *Endoscapes-BBox201* dataset object detection. Faster-RCNN is a two-stage object detector based on Region Proposal Network (RPN) [58] which has become a standard baseline in object detection studies. Based on Faster-RCNN, Cascade-RCNN introduced a multi-stage structure progressively enhanced by IoU thresholds [59]. Unlike RCNN-based models, Deformable-DETR is based on the end-to-end DETR structure, enhanced by utilizing deformable attention mechanism. In Jin’s paper [34], Faster-RCNN was also utilized to compare with 3 winner models [30], [31], [32] of the M2CAI Tool Presence Detection Challenge on frame-level detection of the tools in LC images. The Faster-RCNN and 3 winner models are selected as the benchmark models for the evaluation of *m2cai16-tool-locations* dataset object detection. These benchmark models can cover a broad range of existing architectures, from traditional CNN structure to modern Transformer methods, thereby providing a comprehensive comparison for evaluating our models. We strictly followed the dataset split and evaluator codes in their technical report, paper and open-source repository. The proposed model and the above-mentioned benchmark models were trained and evaluated under the same conditions.

### 3.3 Object position relationship analysis

**Fig. 2:**
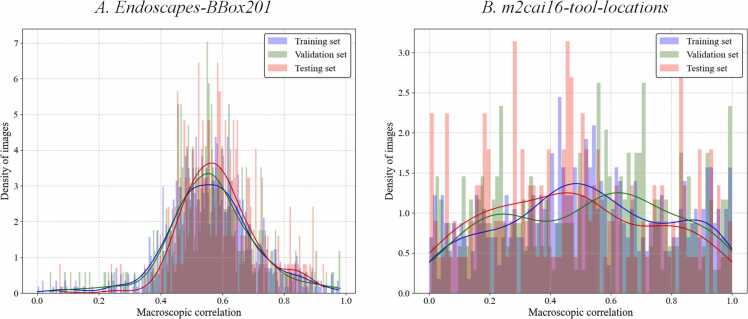
Macroscopic Correlation values of the *A. Endoscapes-BBox201* dataset and *B. m2cai16-tool-locations* dataset.

In this study, we analyzed the position relationship level between the objects of *Endoscapes-BBox201* and *m2cai16-tool-locations* dataset using MC metric. The statistical distribution of MC metrics about those subsets is visualized in Fig. 2. It can be seen from Fig. 2 that the distribution of different subsets (training, validation and testing) is very similar, which indicates the similar statistical characteristics of subsets from the same dataset. As shown in Fig. 2 A, the MC distribution of Dataset A is concentrated around high numerical MC values (>0.5), demonstrating the strong position relationship correlation between objects in this dataset. It suggests that the surgical objects in the LC images, including anatomical structures and tools, are highly correlated in terms of spatial positions. In contrast, Fig. 2B shows that the MC distribution of Dataset B is less concentrated at its peak position. While the peak value remains high (>0.5), the density of images across different MC values is relatively uniform. The reason for this phenomenon is that most images in the dataset contain only fewer tools. This lack of multiple objects in the same image reduces the overall correlation in object positioning, leading to a more evenly distributed MC value density. Utilizing these two datasets provides a comprehensive validation of the proposed method under varying conditions when the datasets exhibit strong or weak positional relationships among objects.

### 3.4 Experiment results analysis

#### 3.4.1 Training characteristics

**Fig. 3:**
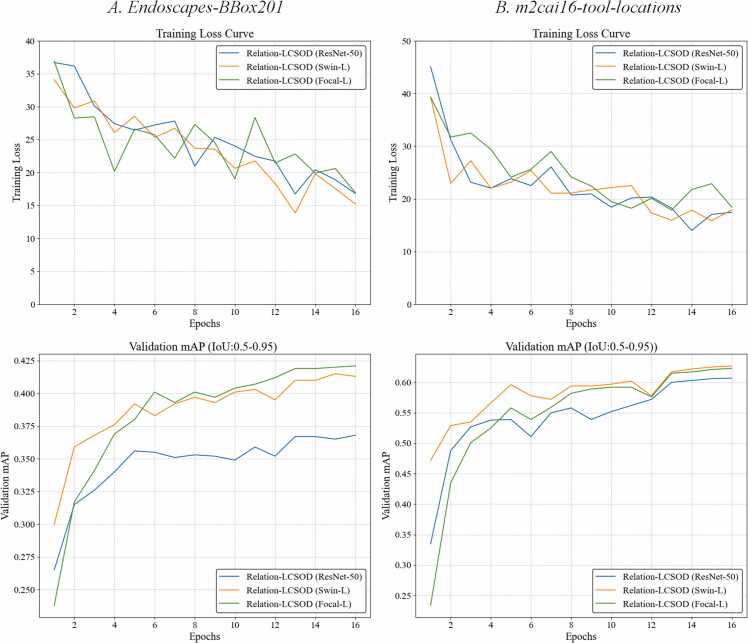
The curves of training loss and validation mAP (IoU:0.50–0.95) for the proposed models in two datasets: *A. Endoscapes-BBox201* dataset and *B. m2cai16-tool-locations* dataset.

During the training process of the proposed model, we observed that the training time is strongly influenced by the complexity of the backbone's architecture. Under the same conditions of batch size, number of workers, learning rate, and image augmentation, fine-tuning the Relation-LCSOD (ResNet-50) model is significantly faster when compared to the other two models. The number of training images in the two datasets is approximately the same, with 1212 training images from the *Endoscapes-BBox201* and 1405 training images from the *m2cai16-tool-locations* dataset. As a result, it took approximately 5 h to finetune the Relation-LCSOD (ResNet-50) model for 16 epochs on each dataset. In contrast, finetuning the Relation-LCSOD (Swin-L) and Relation-LCSOD (Focal-L) for 16 epochs on each dataset required approximately 9 h and 12 h, respectively. Even though the parameters of the encoders, decoders and feed-forward layers are the same, the training time for these three models can be significantly different. Besides, the curves of training loss and overall validation mAP (IoU: 0.5–0.95) are shown in Fig. 3.

As shown in Fig. 3, the convergence speed of the model reflects the different complexity of the two datasets. For Dataset A: *Endoscapes-BBox201*, the validation mAP curve indicates that the performance of models tends to be stable after 12 epochs. However, the Loss curve for training models on Dataset B: *Endoscapes-BBox201* dataset exhibits a convergence pattern that is not entirely smooth. In contrast, models demonstrate a faster and smoother convergence on Dataset B. As shown in both the training Loss curve and validation mAP curve, models can achieve convergence at epoch 8. Besides, the validation mAP values of models on Dataset B (all over 0.60) are generally higher than those of models on Dataset A (all lower than 0.425). From an indirect perspective, it demonstrates that the complexity of Dataset A is higher than that of Dataset B. This is because images from Dataset A contain a greater variety of objects and categories. We also observed that images from Dataset B typically only contain 1 or 2 types of tools at the same time.

#### 3.4.2 Evaluation results on both datasets

**Fig. 4:**
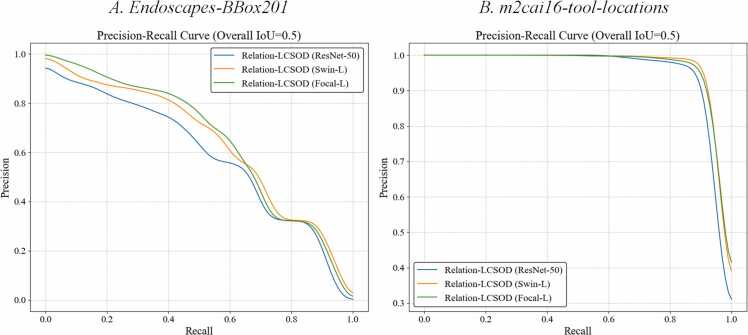
The precision-recall curves for the proposed models’ performance on two datasets at IoU threshold from 50 % to 95 %: *A. Endoscapes-BBox201* dataset and *B. m2cai16-tool-locations* dataset.

After fine-tuning and validation, the proposed models were evaluated on the testing sets of the two datasets. We strictly adhered to the official benchmark testing set splits for both datasets: 312 frames for testing from Dataset A: *Endoscapes-BBox201* and 563 frames from Dataset B. *m2cai16-tool-locations*. As a result, the main evaluation metrics for the two datasets are presented in Table 1, including AP at commonly used IoU thresholds of 50 % and 75 %, and mAP across IoU thresholds from 50 % to 95 %. The Precision-Recall (PR) curves of the models for the two datasets are visualized in Fig. 4, providing a clear graphical representation of the trade-off between precision and recall across IoU thresholds from 50 % to 95 %.

**Table 1:** Evaluation metrics of proposed models’ performance on the Dataset A: *Endoscapes-BBox201* and Dataset B. *m2cai16-tool-locations*, including AP at IoU threshold 50 % (AP@50), AP at IoU threshold 75 % (AP@75) and mAP at IoU threshold from 50 % to 95 % (AP@50:95).

**Models**	**Relation-LCSOD (ResNet-50)**			**Relation-LCSOD (Swin-L)**			**Relation-LCSOD (Focal-L)**		
**Performance**	**AP@50**	**AP@75**	**AP@50:95**	**AP@50**	**AP@75**	**AP@50:95**	**AP@50**	**AP@75**	**AP@50:95**
**Dataset A**									
Cystic Plate	21.9	15.0	10.1	30.8	18.8	14.8	34.0	23.4	17.7
HC Triangle	52.0	43.7	30.0	58.2	53.5	36.8	59.2	56.1	38.3
Cystic Artery	38.7	21.7	15.0	48.5	25.5	18.6	51.9	33.2	23.3
Cystic Duct	58.9	29.1	21.0	59.4	37.2	25.2	60.7	37.3	24.8
Gallbladder	92.5	86.6	75.5	95.4	91.4	80.0	94.1	91.0	79.9
Tool	86.3	82.6	76.1	89.1	86.4	79.8	87.8	85.0	78.7
Overall	58.4	46.5	38.0	63.6	52.1	42.5	64.6	54.3	43.8
**Dataset B**									
Grasper	91.0	81.1	53.7	93.4	85.0	58.4	93.4	83.0	57.2
Bipolar	93.9	78.7	57.7	97.0	87.6	62.3	93.9	84.7	60.2
Hook	100.0	94.5	76.0	100.0	100.0	77.5	100.0	100.0	75.8
Scissors	98.8	89.8	56.4	99.6	93.8	60.0	97.7	93.7	58.8
Clipper	95.2	93.7	70.2	95.3	90.3	70.1	95.1	92.2	68.6
Irrigator	93.6	82.9	50.5	94.5	83.3	52.2	97.5	77.3	49.6
Specimen Bag	92.8	86.9	60.6	95.8	88.7	64.4	97.6	87.5	66.2
Overall	95.0	86.8	60.7	96.5	89.8	63.6	96.5	88.4	62.3

As shown in Table 1, the Relation-LCSOD (Focal-L) model achieves the best overall performance on Dataset A, while the Relation-LCSOD (Swin-L) achieves the best overall performance on Dataset B. It also corresponds to the phenomenon in Fig. 4. As shown in Fig. 4 A, the PR curve of Relation-LCSOD (Focal-L) can generally encompass the other two curves across most recall levels, except for a slight advantage demonstrated by Relation-LCSOD (Swin-L) at recall levels between 0.6 and 0.8. In contrast, as shown in Fig. 4B, the PR curve of Relation-LCSOD (Swin-L) outperforms the other models across all recall levels, demonstrating its consistent dominance on Dataset B. Additionally, the different object categories in Dataset A have a significant impact on the performance of the models. In the results of Dataset A, it can be observed that the AP values of Cystic Plate, Cystic Artery and Cystic Duct are generally lower than that of the other 3 categories, with the AP@50:95 of all models lower than 25 %. On the contrary, the AP values of Gallbladder and Tool are significantly higher than other categories, with the AP@50:95 of all models exceeding 75 %.

#### 3.4.3 Representative samples analysis

**Fig. 5:**
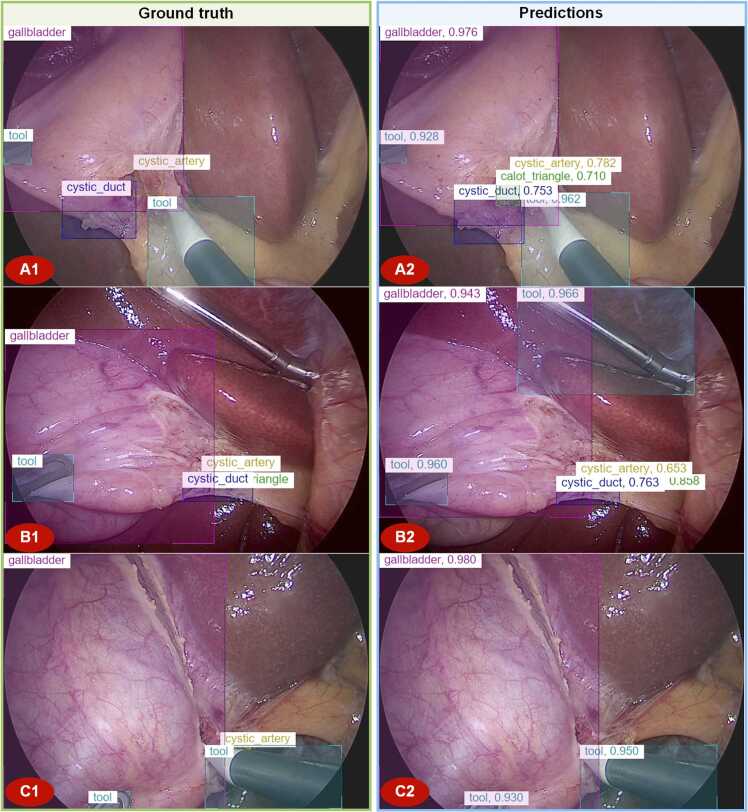
Representative samples from different perspectives in Laparoscopic Cholecystectomy, with ground truth (A1, B1, C1) and predictions (A2, B2, C2) of Relation-LCSOD (Focal-L) for the *Endoscapes-BBox201* dataset.

**Fig. 6:**
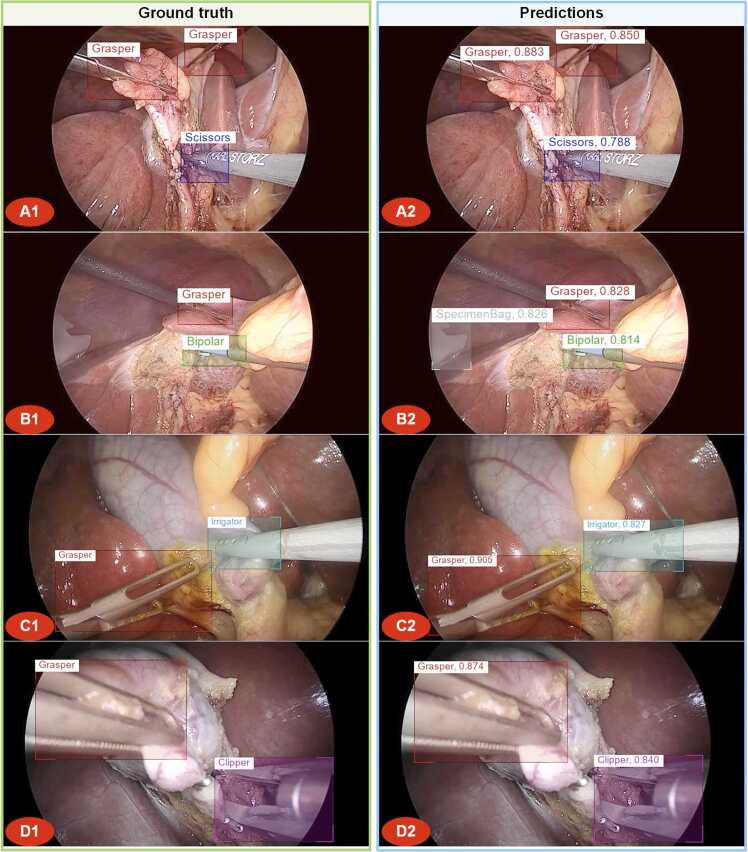
Representative samples with different tools in Laparoscopic Cholecystectomy, with ground truth (A1, B1, C1, D1) and predictions (A2, B2, C2, D2) of Relation-LCSOD (Swin-L) for the *m2cai16-tool-locations* dataset.

To gain insights into the predictions of the model, we examined representative samples from the top-performing models on each dataset. Those samples are presented in Fig. 5, Fig. 6.

Fig. 5 indicates that images in Dataset A typically contain multiple types of objects, including the Gallbladder as the background, Tools as operational objects, and the Cystic Plate, Cystic Artery and Cystic Duct as target objects. Combining the results in Fig. 5 and Table 1, the Gallbladder and Tool categories are easier to detect due to their rich features, such as larger size and distinctive colors. In contrast, the Cystic Plate, HC Triangle, Cystic Artery, and Cystic Duct are relatively more challenging to detect. Despite overall improvements, achieving perfect detection accuracy of surgical tools remains challenging due to the inherently complex nature of biological scenes. In contrast to relatively static domains such as road scenes, surgical endoscopic videos involve highly dynamic environments, characterized by rapid instrument movements (often resulting in motion blur), frequent occlusions, and only partial views of tools. Moreover, surgical tools often share highly similar shapes and appearances, further complicating discrimination. As illustrated in Fig. 5B, for instance, the liver retractor was incorrectly identified as a surgical tool, although it was not annotated as such in the ground truth. Benefiting from the positional relationship analysis of the proposed method, the models can detect the locations of the Cystic artery and Cystic duct based on the spatial position and feature representations of the Gallbladder and tools, even in cases where the predicted bounding boxes have low IoU with the ground truth. Nevertheless, the intricate nature of the laparoscopic surgical environment continues to pose challenges, resulting in occasional failures in accurate recognition. For example, the Cystic Plate is mistakenly detected as the HC Triangle in Fig. 5 A. The models made a false prediction of the HC Triangle because the positions of the HC Triangle often lie between Cystic Artery and Cystic Duct. It is also why the AP of the HC Triangle is significantly higher than that of the Cystic Plate, as its position is more predictable based on its spatial relationships with the Cystic Artery and Cystic Duct. Similarly, in Fig. 5 C, the presence of surgical tools obstructed the field of view, resulting in the missed detection of the Cystic artery. For Dataset B in Fig. 6, representative samples were selected based on the different types of tools present in the images. Since Dataset B only annotates the surgical tools present in the LC images, most images contain only one or two different types of tools at the same time. As a result, the models achieve significantly better performance in detecting tools in Dataset B. Combining the results in Fig. 6 and Table 1, the proposed models successfully detect all different types of tools in Dataset B.

#### 3.4.4 The effect of postprocessing step

In this study, a postprocessing step was introduced to enhance the suitability of models for detecting surgical objects in LC images. During the experiments, we observed that the number of predicted bounding boxes was still higher than the actual number of bounding boxes in the ground truth. The number of predicted bounding boxes could exceed 10 times the actual number of bounding boxes in the ground truth. To address this issue and enhance the suitability of the proposed models for surgical object detection in LC images, a more efficient postprocessing method was implemented. For Dataset A: *Endoscapes-BBox201*, predictions were filtered as follows: for categories Cystic Plate, HC Triangle, Cystic Artery, Cystic Duct, and Gallbladder, only the predictions with the highest scores were retained. For the Tool category, the top two predictions with the highest scores were kept. Similarly, in Dataset B: *m2cai16-tool-locations*, the top two highest-scoring predictions were kept for Grasper. For all other categories, only the predictions with the highest confidence scores were retained.

The effect of adding this postprocessing step is summarized in Table 2. We selected and presented the model with the best performance for each dataset: Relation-LCSOD (Focal-L) for Dataset A and Relation-LCSOD (Swin-L) for Dataset B.

**Table 2:** The effect of adding the postprocessing step to the proposed model, including the reduction of the predicted boxes and the change of the AP. Ground truth: number of true bounding boxes in testing sets. Predictions (before/after): number of predicted bounding boxes by the proposed models before/after the postprocessing step. Percentages: changing percentages of the AP@50:95 after the postprocessing step.

**Results**	**Ground truth**	**Predictions (before)**	**Predictions (after)**	**AP@50:95 (before)**	**AP@50:95 (after)**	**Percentages**
**Dataset A**						
Cystic Plate	130	16,290	291	20.6	17.7	−14.08 %
HC Triangle	124	7508	291	40.7	38.3	−5.90 %
Cystic Artery	192	17,127	291	26.1	23.3	−10.73 %
Cystic Duct	258	23,895	312	28.8	24.8	−13.89 %
Gallbladder	285	3172	312	82.7	79.9	−3.39 %
Tool	496	25,608	624	82.8	78.7	−4.95 %
Overall	1485	93,600	2121	47.0	43.8	−6.81 %
**Dataset B**						
Grasper	293	129,731	1126	59.0	58.4	−1.02 %
Bipolar	95	3707	561	62.9	62.3	−0.95 %
Hook	64	11,757	563	77.4	77.5	+0.13 %
Scissors	84	1050	549	60.0	60.0	+0.00 %
Clipper	64	2542	559	70.4	70.1	−0.43 %
Irrigator	84	8755	560	52.4	52.2	−0.38 %
Specimen Bag	96	11,204	559	66.6	64.4	−3.30 %
Overall	780	168,746	4477	64.1	63.6	−0.78 %

As shown in Table 2, the postprocessing step effectively reduced the number of predicted bounding boxes. For Dataset A, the total number of predictions was reduced from 93,600 to 2121, which is much closer to the ground truth count of 1485. Similarly, for Dataset B, the total number of predictions was reduced from 168,746 to 4477, significantly narrowing the gap toward the ground truth number. With the implementation of the postprocessing step, the results of the proposed models are more closely aligned with the clinical situation. At the same time, the performance decrease remained within an acceptable range, ensuring sufficient accuracy and efficiency for practical use in surgical object detection. For Dataset A, the overall mAP at IoU thresholds from 50 % to 95 % decreased by 6.81 %, while the decrease was much smaller for Dataset B, with only 0.78 %. It indicates that the postprocessing step had a more significant impact on the performance of models for Dataset A, due to the more complexity of the objects and position relations in the images. However, the performance reduction for both datasets remains within an acceptable range, demonstrating the effectiveness and accuracy of the proposed models after the postprocessing step. With the postprocessed results, the proposed models can be utilized for deeper clinical analysis and research, such as the CVS prediction [27] and operative skill assessment [34].

#### 3.4.5 Comparison analysis on the Endoscapes-BBox201 dataset

To validate the accuracy and effectiveness of the proposed models, we conducted a comparative analysis of the evaluation results versus benchmark models on the *Endoscapes-BBox201* dataset. Faster-RCNN [58], Cascade-RCNN [59] and Deformable-DETR [60] were selected as benchmark models for object detection of the dataset. The proposed models were trained, validated, and tested on the same image sets as the benchmark models to ensure a fair comparison. Additionally, we employed the same evaluator codes from the open-source repository (https://github.com/CAMMA-public/SurgLatentGraph/tree/main/evaluator) to avoid any potential calculation bias. The mAP values at IoU thresholds from 50 % to 95 % of both the proposed models and benchmark models on the *Endoscapes-BBox201* dataset are presented in Table 3. As shown in Table 3, the Deformable-DETR outperforms all other benchmark models, achieving the best mAP at all classes. However, all proposed models can outperform the Deformable-DETR at all classes. The proposed Relation-LCSOD model with Swin-L and Focal-L backbones can significantly outperform the Deformable-DETR in overall performance with improvements of 29.97 % and 33.95 % respectively. With relatively lower computational power consumption, the Relation-LCSOD (ResNet-50) still achieves a 16.21 % overall improvement. As for different classes, it can be observed that the detection performance for anatomical landmarks, including Cystic Plate, HC Triangle, Cystic Artery, and Cystic Duct, was generally lower than that for Gallbladder and Tool. These anatomical landmarks are often partially occluded or covered by surrounding tissue. In addition, substantial variability arising from tissue characteristics, patient-specific anatomy, and case diversity further complicates both the annotation and detection processes. Even the clinical annotation of anatomical structures is subject to considerable interobserver variability. Importantly, despite these challenges, the proposed model achieved significant improvements in the detection accuracy of these structures. Especially for Cystic Plate and HC Triangle, the proposed models can significantly improve the detection AP. The Relation-LCSOD (Focal-L) model surpasses the Deformable-DETR by 90.32 % on Cystic Plate and 92.46 % on HC Triangle, and even achieves more than double the performance compared to Faster-RCNN and Cascade-RCNN.

**Table 3:** Results comparison with the benchmark models on the *Endoscapes-BBox201* dataset. The highest detection mAP for each class and the proposed models are highlighted in bold. The improvement percentage was calculated by comparing the proposed models versus the best benchmark model, Deformable-DETR.

**Detection AP and mAP (IoU@50:95)**							
	**Cystic Plate**	**HC Triangle Dissection**	**Cystic Artery**	**Cystic Duct**	**Gallbladder**	**Tool**	**Overall**
Faster-RCNN [58]	8.0	19.4	12.6	15.5	62.9	61.5	30.0
Cascade-RCNN [59]	7.5	18.4	12.1	13.8	61.5	62.4	29.3
Deformable-DETR [60]	9.3	19.9	14.9	19.0	69.6	63.7	32.7
Relation-LCSOD (ResNet−50)	10.1 (+8.60 %)	30.0 (+50.75 %)	15.0 (+0.67 %)	21.0 (+10.53 %)	75.5 (+8.48 %)	76.1 (+19.47 %)	38.0 (+16.21 %)
Relation-LCSOD (Swin-L)	14.8 (+59.14 %)	36.8 (+84.92 %)	18.6 (+24.83 %)	**25.2** **(+32.63 %)**	**80.0** **(+14.94 %)**	**79.8** **(+25.27 %)**	42.5 (+29.97 %)
Relation-LCSOD (Focal-L)	**17.7** **(+90.32 %)**	**38.3** **(+92.46 %)**	**23.3** **(+56.38 %)**	24.8 (+30.53 %)	79.9 (+14.80 %)	78.7 (+23.55 %)	**43.8** **(+33.95 %)**

#### 3.4.6 Comparison analysis on the m2cai16-tool-locations dataset

Similarly, a comparative analysis between the proposed models versus benchmark models was conducted on the *m2cai16-tool-locations* dataset. Unlike the *Endoscapes-BBox201* dataset, the objects from the *m2cai16-tool-locations* dataset do not exhibit strong position relations. Evaluating the performance of the proposed models on this dataset can sufficiently demonstrate the effectiveness of the models in different situations Four benchmark models [30], [31], [32], [34] were proposed and tested on the *m2cai16-tool-locations* dataset. The proposed models were trained, validated, and tested on the same image sets as the benchmark models to ensure a fair comparison. Based on the paper of Jin et al. [34], the mAP values at IoU thresholds of 50 % of both the proposed models and benchmark models on the *m2cai16-tool-locations* dataset are presented in Table 4. As shown in Table 4, the model of Jin et al. achieves the best performance among all benchmark models. However, all proposed models can outperform the model of Jin et al. The proposed models improve the overall mAP by 16.14 %, 17.97 %, and 17.87 % respectively, with smaller differences across the various backbones. The improvement percentages of the proposed models are not that significant on the *m2cai16-tool-locations* dataset. The improvements for detecting most categories range from 4 % to 30 %, except for the detection of Scissors, which shows a significant improvement of up to 40.68 %. Additionally, both the Relation-LCSOD (Swin-L) and Relation-LCSOD (Focal-L) achieve the best overall performance, while the Relation-LCSOD (Swin-L) even acquires higher mAP across more categories, such as Bipolar, Scissors and Clipper. Considering the training time for both models, with 9 h for Swin-L backbone and 12 h for Focal-L backbone, the Relation-LCSOD (Swin-L) is proven to be more suitable for less complex dataset.

**Table 4:** Results comparison with the benchmark models on the *m2cai16-tool-locations* dataset. The highest detection mAP for each class and the proposed models are highlighted in bold. The improvement percentage was calculated by comparing the proposed models versus the best benchmark model, the model of Jin et al.

**Detection AP and mAP (IoU@50)**								
	**Grasper**	**Bipolar**	**Hook**	**Scissors**	**Clipper**	**Irrigator**	**Specimen Bag**	**Overall**
Twinanda et al. [30]	82.2	73.9	89.4	17.0	43.6	12.5	72.2	52.5
Sahu et al. [32]	73.9	40.8	95.1	26.2	35.3	33.2	76.6	54.4
Raju et al. [31]	NA	NA	NA	NA	NA	NA	NA	63.7
Jin et al. [34]	87.2	75.1	95.3	70.8	88.4	73.5	82.1	81.8
Relation-LCSOD (ResNet−50)	91.0 (+4.36 %)	93.9 (+25.03 %)	**100.0** **(+4.93 %)**	98.8 (+39.55 %)	95.2 (+7.69 %)	93.6 (+27.35 %)	92.8 (+13.03 %)	95.0 (+16.14 %)
Relation-LCSOD (Swin-L)	**93.4** **(+7.11 %)**	**97.0** **(+29.16 %)**	**100.0** **(+4.93 %)**	**99.6** **(+40.68 %)**	**95.3** **(+7.81 %)**	94.5 (+28.57 %)	95.8 (+16.69 %)	**96.5** **(+17.97 %)**
Relation-LCSOD (Focal-L)	**93.4** **(+7.11 %)**	93.7 (+24.77 %)	**100.0** **(+4.93 %)**	97.7 (+37.99 %)	95.1 (+7.58 %)	**97.5** **(+32.65 %)**	**97.6** **(+18,88 %)**	**96.5** **(+17.97 %)**

## 4 Discussion

This study introduced the Relation-LCSOD model with three backbones to effectively address the challenges of detecting surgical objects in LC images. Two widely used public datasets: A. *Endoscapes-BBox201* dataset and B. *m2cai16-tool-locations* dataset, were selected to validate the accuracy, robustness and effectiveness of the proposed models. The MC analysis results revealed the distinct differences in the position relationship strength between the two datasets. Dataset A exhibited strong position relations and Dataset B showed comparatively weaker position relations. The utilization of these two datasets enables comprehensive validation of the proposed models across diverse situations. We strictly adhered the official data split and employed the provided evaluator to guarantee a fair and unbiased comparison between the proposed models and the benchmark models for both datasets. The experimental results demonstrate the accuracy and effectiveness of the proposed models on both datasets. In the proposed model, the position relation encoder and refined progressive attention mechanism can effectively analyze and utilize the position relation information among objects in the LC image. The parallel relation streaming pipeline can effectively address the insufficient positive supervision and duplication removal in object detection. Additionally, a postprocessing step can significantly reduce redundant bounding boxes by over 90 %, aligning the predictions more closely with clinical reality. To enhance the practical utility of our work and support its adoption by clinicians and endoscopists, we developed a user-friendly web-based application, LCSOD-tool. This tool enables users to upload LC images and automatically perform surgical object detection using our models. The application has been published on GitHub for ease of use and further development (https://github.com/xyn-abc/LCSOD-tool). With the LCSOD-tool as a foundation, real-time intraoperative deployment could become feasible through the resolution of remaining infrastructural and regulatory requirements.

The key findings from the experiments are summarized as follows. The proposed models demonstrated significant performance improvements on the *Endoscapes-BBox201* dataset, especially for objects with challenging detectability, such as Cystic Plate, HC Triangle, Cystic Artery, and Cystic Duct. The Relation-LCSOD (Focal-L) model outperformed the leading benchmark model, Deformable-DETR, achieving an overall mAP improvement of 33.95 %. For Cystic Plate and HC Triangle, the detection AP improved by 90.32 % and 92.46 %, respectively. The results demonstrated the ability of the proposed models to effectively capture and analyze the position relation information among different objects, enhancing the detection performance on objects with subtle or unclear features within complex multi-object contexts. However, due to the indistinct features and lack of clear positional relevance to other objects, model performance on the Cystic Plate remains comparatively lower and needs further improvement.

For the *m2cai16-tool-locations* dataset, the proposed models also demonstrated effective performance, improving the overall mAP by up to 17.97 % compared to benchmark models. In contrast to the *Endoscapes-BBox201* dataset, this dataset presents a more evenly distributed MC value density, assessing the generalization ability of the proposed models. The comparison results revealed that the improvement percentages of the proposed models were relatively modest, demonstrating improvements with a 4–30 % range for most tool categories. The improvement is constrained by the limited utilization of position relation information among objects in the *m2cai16-tool-locations* dataset. However, the proposed models consistently outperformed the benchmark models, validating the robustness of the proposed models across varing conditions.

Considering the influence of different backbones, the Relation-LCSOD (Swin-L) achieved a balanced trade-off between performance and training efficiency, requiring only 9 h for training compared to 12 h for the Focal-L backbone. Especially on simpler datasets such as the *m2cai16-tool-locations*, the accuracy difference between Swin-L and Focal-L backbone is not obvious. It proves the suitability of Swin-L for situations requiring computational efficiency. Focal-L remains the preferred backbone for more complex datasets like the *Endoscapes-BBox201*.

The findings in this study suggest that object complexity, position relationships, and dataset characteristics are important factors influencing the performance of the proposed models. By integrating position relation analysis and postprocessing, the proposed models not only enhance the detection accuracy on LC datasets but also align more closely with clinical expectations. The proposed models are trustworthy and capable of further clinical research. The proposed LCSOD-tool can offer real-time assistance and help decision-making for clinicians and endoscopists. Such advancements could ultimately lead to the improvement of patient outcomes through better surgical interventions and more accurate clinical assessments. However, the detection of objects with unclear features or limited position context remains challenging, such as the Cystic Plate. Future work could aim to expand the proposed models with multi-center datasets that capture greater variability in surgical equipment, anatomic structure and imaging conditions. In addition, utilizing temporal information from surgical videos, such as instrument motion patterns and temporal consistency, may further enhance model performance and generalizability across diverse clinical settings.

## 5 Conclusion

In the study, a Relation-LCSOD model was proposed based on existing structures for surgical object detection in LC images. The experiment results on two datasets demonstrated significant performance improvements of the models, especially in detecting anatomically relevant objects. By integrating position relation analysis and effective postprocessing steps, the model can promote trust in AI-driven clinical research and align predictions closely with clinical realities. Based on the models, future work could focus on more clinical applications, such as CVS prediction and operation skill assessments. The study advances the trusted integration of AI into medical imaging workflows and supports its practical application into clinical practice.

## CRediT authorship contribution statement

**Christiane Bruns:** Supervision, Funding acquisition, Project administration. **Hans Fuchs:** Supervision, Project administration, Funding acquisition, Resources. **Jennifer Eckhoff:** Writing – review & editing, Supervision, Project administration, Conceptualization, Data curation, Investigation, Resources. **Yutong Ban:** Writing – review & editing, Resources, Conceptualization, Data curation, Investigation. **Yinan Xu:** Writing – original draft, Visualization, Validation, Methodology, Investigation, Conceptualization, Software, Writing – review & editing. **Dolores Krauss:** Writing – review & editing, Project administration, Resources. **Yue Zhao:** Writing – review & editing, Project administration.

## Conflict of Interest

The authors declare that there are no conflicts of interest in this study.
